# Exploring the Potential Effects and Mechanisms of *Asarum sieboldii* Radix Essential Oil for Treatment of Asthma

**DOI:** 10.3390/pharmaceutics14030558

**Published:** 2022-03-03

**Authors:** Jae Min Han, Mi Hye Kim, La Yoon Choi, Gyeongsang Kim, Woong Mo Yang

**Affiliations:** Department of Convergence Korean Medical Science, College of Korean Medicine, Kyung Hee University, 26 Kyungheedae-ro, Dongdaemun-gu, Seoul 02447, Korea; jmhan77@khu.ac.kr (J.M.H.); kimmihye526@khu.ac.kr (M.H.K.); lydanas@khu.ac.kr (L.Y.C.); rlarudtkd506@khu.ac.kr (G.K.)

**Keywords:** air pollution, *Asarum sieboldii*, asthma, essential oil, particulate matter

## Abstract

Asthma, a common chronic pulmonary disorder characterized by airway remodeling, hyperresponsiveness and obstruction, can be aggravated by repeated exposure to particulate matter (PM). The potential effect and mechanisms of *Asarum sieboldii* Radix essential oil (AEO) against asthma were explored based on network pharmacology. AEO was pre-treated using a nebulizer for 3 weeks and the mice were sensitized to ovalbumin (OVA) and PM_10_ with the co-treatment of AEO for 4 weeks. In addition, A549 lung epithelial cells were sensitized with PM_10_ to investigate the underlying mechanisms of AEO regarding the lung-fibrosis-related mediators. The target genes of methyl eugenol, a main compound of AEO, were highly matched by 48% with the gene set of asthma. AEO markedly inhibited the increase in epithelial thickness through the accumulation of goblet cells in the airways. Collagen deposition in the lung tissues of OVA+PM_10_-challenged asthmatic mice was significantly decreased by AEO. AEO also inhibited the influx of inflammatory cells in the bronchoalveolar lavage fluid, as well as the increases in serum IgE and IgG_2a_ and cytokines in the lung tissues. Furthermore, AEO regulated the expressions of fibrotic mediators, especially POSTN and TGF-β. In conclusion, we expect that AEO can be one of the effective alternative therapeutics to relieve asthma.

## 1. Introduction

Air pollution, a major environmental health issue in global regions, causes a variety of symptoms, diseases and even mortality and is a social and economic burden [1]. It is well established that exposure to air pollution aggravates airway inflammation via modulation of airway epithelium and production of inflammatory cytokines [2]. Especially, asthma is defined by a common chronic disorder of the airways involving the complicated interactions of airflow limitation, airway hyperresponsiveness and airway inflammation [3]. It is announced to threaten human health, as 300 million people are affected by asthma globally, to which 100 million would be expected to be added by 2025 [4]. Airborne particulate matter (PM) is a one of major air pollutants causing harmful effects on the human respiratory condition [5]. In both outdoor and indoor conditions, exposure to PM promotes adverse effects in the respiratory system and other organs, contributing to the prevalence of pulmonary diseases; in particular, PM_10_ is known as a major inducing and exacerbating factor of pediatric asthma, the most common chronic pulmonary disease in children [6].

Airway inflammation, a defense mechanism against pathogens that involves the production and activation of inflammatory cells, cytokines, immunoglobulins and other factors, is a typical characteristic of pulmonary diseases, implicating an impaired airway equilibrium and airway structural changes [7]. As a consequence of the inflammation and wound repair after injury, airway remodeling, including accumulation of the extracellular matrix (ECM), thickening of the airway wall and mucin-secreting goblet cell metaplasia, contributes to airflow limitation and obstruction, which are critical manifestations in asthma [8]. Inhalation of corticosteroids, short-acting β2-agonists and medication of budesonide/formoterol is typically recommended in asthmatic adults and adolescents. However, risk of exacerbations, side effects and resistance to inhalants and medication remain unmet needs for patients with asthma [9]. Recently, the inhalation of essential oils, also called ‘aromatherapy’, has been treated as a relieving remedy in terms of alternative and complementary medicine. In both Eastern and Western countries, many studies have been conducted on the effects of the application of essential oil inhalation on allergic and upper airway diseases [10]. Experimentally, Ueno-lio et al. reported the suppressive effect of inhaled Lavender essential oil on allergic airway inflammation and mucous-cell hyperplasia in the murine model with asthma [11]. Essential oils of *Salvia limbate*, which include various chemicals such as spathulenol, caryophyllene oxide, bicyclogermacrene, germacrene D, (E)-caryophyllene, linalool, (Z)-β-ocimene, 1,8-cineole and α-pinene, have been reported to have anti-oxidant and anti-bacterial activities [12,13]. However, attempts to find therapeutic potentials against asthma using essential oil are still rarely conducted at the experimental research level.

*Asarum sieboldii* radix, called seshin in Korean, saishin in Japanese and Xixin in Chinese, is a commonly used medicinal herb in Asian countries [14]. In Korean and Chinese traditional medicine, some decoctions including *Asarum sieboldii* radix have been used to treat pulmonary symptoms and pain such as cough, asthma, allergies, chronic bronchitis, toothache, headache and aphthous stomatitis [15,16,17]. In addition, the essential oil of *Asarum sieboldii* radix (AEO) has been also reported to have anti-fungi, anti-allergic and acaricidal effects [18,19]. Methyl eugenol, a main component of AEO, as well as an essential oil derived from clove buds, cinnamon, tulsi, turmeric, pepper and ginger, has been known as a volatile flavor compound and perfumery [20]. Recently, it has been extensively used for aromatherapy as an essential oil [20,21]. Taken together, the ameliorative effects of AEO containing methyl eugenol on the respiratory system could be inferred based on the traditional use, ingredients and research.

Now, this study investigated the effects and molecular mechanism of AEO on asthma through a network pharmacological analysis and experimental verification in vivo and in vitro. The inhibitory effects of AEO inhalation against asthma were analyzed by observing the histology of the lung and trachea in an established ovalbumin (OVA) and PM_10_-treated murine model, as well as in the PM_10_-treated airway A549 epithelial cells. Cell numbers, including macrophages, neutrophils, eosinophils and lymphocytes, in bronchoalveolar lavage fluid (BALF) were counted and the serum immunoglobulin (Ig)E and IgG2a levels were analyzed. In addition, the expressions of pro-inflammatory cytokines, pro-fibrotic factors and fibro-genetic factors of epithelial–mesenchymal transition (EMT) markers were confirmed in the lung tissues and PM_10_-treated A549 cells.

## 2. Materials and Methods

### 2.1. Network Pharmacological Analysis

The network of AEO was constructed based on the target gene of methyl eugenol, because methyl eugenol is well known to be a main compound of AEO [22]. The chemical gene co-occurrences of methyl eugenol (PubChem CID 7127) in the literature were collected from the PubChem open database (https://pubchem.ncbi.nlm.nih.gov 24 February 2022). A total of 100 genes formed the AEO network, as found using Cytoscape String App. To compare the association between AEO and asthma, genes related to asthma were gathered from the GeneCards open database (http://www.genecards.org/ 24 February 2022). In total, 7535 genes related to asthma were collected and compared with the gene set of AEO. The overlapping genes were counted.

### 2.2. Preparation of AEO

*A*. *sieboldii* radix was purchased from DYHERB Corp. (Seoul, Korea). A total of 100 g of dried *A. siebodii* radix was placed in a round flask and connected to a water flask and condenser. A liter of water was poured in the water flask and boiled. The steam crossed the *A. siebodii* radix and charged with floral water and essential oil in the condenser. Following the decantation of the tube with charged floral water and essential oil, AEO was separated by centrifugation at 4000 rpm for 10 min. Eventually, whole AEO was extracted for 6 h by the hydrodistillation method. The yield of AEO was 1.2 mL/100 g (*v/w*%) and the moisture of AEO was 99.7%. AEO was kept at 4 °C until use. To identify the components of AEO, a gas chromatography/mass spectrometry (GC-MS) analysis was conducted using a GC-2010 shimadzu and a GCMS-QP2010Plus with an Agilent DB-5 (30 m × 0.25 mm; 0.25 μm) column. The program of GC was 100 °C column oven temperature, 130 °C injection temperature and He carrier gas with 0.66 mL/min flow, as well as 200 °C ion source temperature and 300 °C interface temperature, with a 100–500 *m*/*z* scan range. The result is shown in Figure S1 (Supplementary material).

### 2.3. Animal Treatment

Five-week female BALB/c mice were purchased from Raonbio Inc. (Yongin, Korea). Mice were housed in a controlled facility with constant temperature and humidity and a 12 h light/dark cycle for a week. All animal procedures were approved by the Committee on Care and Use of Laboratory Animals of the Kyung Hee University (KHUASP(SE)-19-098; Seoul, Korea). Mice were randomly divided into 5 groups (*n* = 6), i.e., (1) normal mice (NOR); (2) asthmatic mice challenged with OVA and PM_10_ and nebulized with normal saline (OVA+PM_10_); (3) asthmatic mice challenged with OVA and PM_10_ and nebulized with 2 mg/kg (calculated as 0.6%) dexamethasone (DEX); (4) asthmatic mice challenged with OVA and PM_10_ and nebulized with 0.0002% of AEO (AEO 0.0002); and (5) asthmatic mice challenged with OVA and PM_10_ and nebulized with 0.02% of AEO (AEO 0.02). All samples including saline, DEX and AEO were administered by nasal inhalation by a nebulizer (Philips, Amsterdam, The Netherlands) 3 times per week for 5 min through 7 weeks of the whole experiment. After 3 weeks of inhalation of the samples, each group was immunized with 100 mg OVA (Sigma-Aldrich Inc., St. Louis, MO, USA) and 5 mg aluminum hydroxide AlOH3 (Sigma-Aldrich Inc., St. Louis, MO, USA) in 1 mL of saline by intraperitoneally injection at intervals of 1 week for 3 weeks, on the days 21, 28 and 35. After immunization, the mice were intranasally challenged with 1 mg OVA and 100 μg of PM_10_ in 50 μL of normal saline per mice on days 46–48. The samples were continued to be treated for the whole 7 weeks of the experiment.

### 2.4. Histological Analysis

On day 49, the mice were sacrificed under anesthesia with the injection of avertin (Sigma-Aldrich Inc., St. Louis, MO, USA) and lung tissues were dissected and immersed in 10% neutralized formalin for 24 h. Following tissue fixation, lung tissues were washed and dehydrated by the series concentration gradient of ethanol and xylene for embedding in a paraffin block. The blocks were sectioned at 4 μm thickness and stained by hematoxylin and eosin (H&E), periodic acid–Schiff (PAS) and Masson’s trichrome solution. Tissue slides were visualized with Leica Application Suite (LAS) Microscope Software (Leica Microsystems Inc., Wetziar, Land Hessen, Germany) to determine the histological structure, collagen deposition and reaction of mucin.

### 2.5. Cell Counting in BALF

The lungs of each mouse under anesthesia were washed with 1 mL of buffered saline. The collected BALF was centrifuged at 4000 rpm for 30 min. The supernatant of BALF was stained with trypan blue dye in a hemocytometer and monitored under a microscope. Cells, including macrophages, neutrophils, eosinophils and lymphocytes, were counted with evaluation per slide in triplicate.

### 2.6. Enzyme-Linked Immunosorbent Assay

After sacrifice, blood samples were collected by retro-orbital sinus and centrifuged to obtain serum. Serum IgE and IgG_2a_ were analyzed by each commercial enzyme-linked immunosorbent assay (ELISA) kit (Cat. No. #555248 and #552576 BD OptEIA™; BD Biosciences, Franklin Lakes, NJ, USA).

### 2.7. Cell Treatment

A549 cells, human lung epithelial cells, were purchased from Korean Cell Line Bank (Seoul, Korea). Cells were cultured in Dulbecco’s modified Eagle medium (DMEM) (Gibco; Thermo Fisher Scientific, Inc., Waltham, MA, USA) supplemented with 10% *v*/*v* fetal bovine serum (Gibco), 2 mM glutamine, 100 IU/mL penicillin and 100 μg/mL streptomycin (Gibco) at 37 °C in a 5% CO_2_ culture chamber, which maintained homeostasis in the mammalian culture system. AEO was blended with a 1:10 ratio of dimethyl sulfoxide (DMSO) as a stock solution (100 μL/mL) and kept at −20 °C until use. A549 cells were treated with 1 μM DEX, AEO 10^−3^, 10^−2^ and 10^−1^ μL/mL blended with 100 μg/mL of PM_10_. For the monitoring of the A549 cells’ metabolic activity by AEO treatment, A549 cells were seeded in a 96-well plate at 1 × 10^4^ cells/well. AEO 10^−3^, 10^−2^ and 10^−1^ μL/mL dissolved in DMSO were treated for 24 h. A total of 2 mg/mL of 3-(4,5-dimethyl-2-thiazolyl)-2,5-diphenyltetrazolium bromide (MTT) (Invitrogen™, Waltham, MA, USA) was added at 50 μL/well for 2 h until crystal formazan production. For eluting formazan crystals, absolute DMSO was dissolved at 50 μL/well and incubated for 30 min. The optical density was measured at 570 nm. Non-treated A549 cells showed a cobblestone-like cell shape, whereas spindle-like or fibroblast-like cells were observed in the PM_10_-exposed A549 cells. Treatment of DEX and AEO with the concentrations of 10^−6^, 10^−5^ and 10^−4^ (*v*/*v*%) showed to be as normal as the original epithelial phenotype of cell shape (Figure S2). In addition, the cell viability was not changed by 10^−6^, 10^−5^ and 10^−4^ (*v*/*v*%) AEO treatments (Figure S3).

### 2.8. Reverse Transcription Polymerase Chain Reaction (RT-PCR)

Total ribonucleic acid (RNA) was isolated from lung tissues and A549 cells by TRIzol reagent (Invitrogen™). Homogenates were separated by chloroform and incubated for 5 min at room temperature (RT). After centrifugation, isopropanol was added the same amount of supernatant of homogenates. The mixtures were gently inverted, incubated for 3 min at RT and centrifuged at 17,000 rpm for 15 min. The total RNA pellets were washed with 70% ethanol and air-dried for 5–10 min. RNA pellets were dissolved in RNase free water. The cDNA was synthesized with 1 μg of total RNA using a Maxime RT PreMix kit (iNtRON Biotechnology, Inc.) (Seongnam, Republic of Korea). By using specific primers, such as interleukin (IL)-4, IL-13, periostin (POSTN), transforming growth factor (TGF)-β, tumor necrosis factor (TNF)-α, IL-1β, IL-6, collagen type I alpha 1 (COL1A1) and collagen type III alpha 1 (COL3A1) in a Maxim PCR premix kit (iNtRON Biotechnology, Inc.), complementary DNA (cDNA) was amplified. The PCR was performed for 30 s of denaturation at 94 °C, 1 min of annealing at 52–62 °C and 1 min of extension at 72 °C. The amplified products were analyzed with gel electrophoresis by 2% agarose gel. The expression levels of specific RNAs were detected by using a gel documentation system (DAIHAN; Daegu, Korea) and were quantified to GAPDH.

### 2.9. Protein Immunoblot Analysis

By using radioimmunoprecipitation assay (RIPA) buffer with protease inhibitor cocktails, total proteins were extracted in lung tissue and A549 cells. The protein concentrations of lysates were determined by the Bradford assay. Protein lysates were denatured at 98 °C for 5 min and loaded at 10 μg/lane in 7.5% polyacrylamide gel. Separated linear proteins were transferred onto polyvinylidene fluoride (PVDF) membranes and blocked with 3% bovine serum albumin (BSA) at RT for 1 h. Specific primary antibodies (1:1000), which were snail (Cell signaling, MA, USA; #3879S), E-cadherin (Cell signaling; #3195S), N-cadherin (Cell signaling; #4061S), vimentin (Cell signaling; #571S) and fibronectin (BD bioscience, NJ, USA; #610078), diluted in tris-buffered saline with 0.1% Tween 20 (TBS-T), were incubated at 4 °C overnight. The secondary antibodies anti-mouse (Santa Cruz Biotechnology, TX, USA; #sc-516102) and anti-rabbit (Santa Cruz Biotechnology; #sc-2357) diluted in TBS-T were incubated at RT for 1 h. Membranes were incubated with enhanced chemiluminescence (ECL) substrate and detected by a chemiluminescence imaging system (Young In, Seoul, Korea). The visualized bands were quantified using the computerized densitometry system Image J (NIH, Bethesda, MD, USA).

### 2.10. Statistical Analysis

All results are indicated as the means ± standard error of the mean (S.E.M.). For comparison with data, a one-way ANOVA followed by Tukey’s multiple comparison tests was used in these groups. The *p*-values considered statistically significant were given as follows: * *p* < 0.05, ** *p* < 0.01, *** *p* < 0.001.

## 3. Results

### 3.1. Gene Comparison with AEO and Asthma

**Figure 1:**
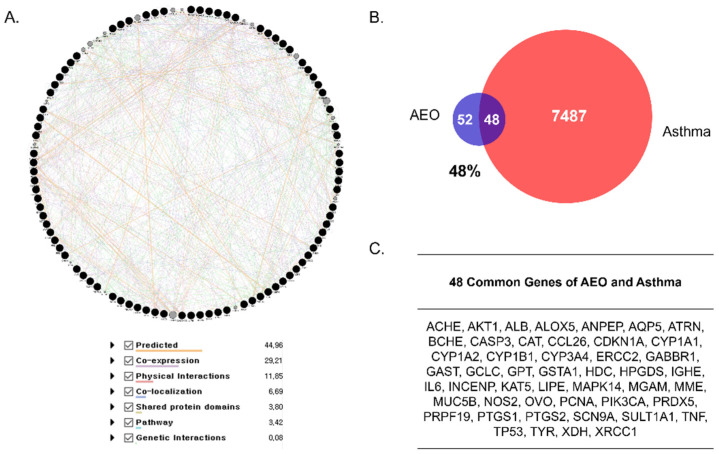
Network pharmacological analysis of AEO. (**A**) Network of AEO with 106 nodes and 968 edges. The nodes and edges indicate the targeted genes of AEO and their relationships, respectively. The black nodes present seed nodes and the gray ones are nodes that interact with the seed nodes. Yellow, predicted; purple, co-expression; pink, physical interactions; blue, co-localization; light green, shared protein domains; sky, pathways; green, genetic interactions. (**B**) Veen diagram of intersection targets between AEO network and the gene sets of asthma disease. (**C**) Common genes of AEO and asthma.

The network of AEO was created with 100 target genes of AEO. A total of 106 nodes and 968 edges formed the AEO network, showing interconnectivity either by reported predicted, co-expression, physical interactions, co-localization, shared protein domains, pathways or genetic interactions (Figure 1A). Based on this network, we investigated the correlation between AEO and asthma via comparison of each contained gene. The gene set of asthma including 7535 genes was compared with that of AEO derived from methyl eugenol. The network of AEO had 48 intersecting genes compared to the whole 100 genes of asthma, showing a 48% match (Figure 1B). The overlapping genes were *ACHE, AKT1, ALB, ALOX5, ANPEP, AQP5, ATRN, BCHE, CASP3, CAT, CCL26, CDKN1A, CYP1A1, CYP1A2, CYP1B1, CYP3A4, ERCC2, GABBR1, GAST, GCLC, GPT, GSTA1, HDC, HPGDS, IGHE, IL6, INCENP, KAT5, LIPE, MAPK14, MGAM, MME, MUC5B, NOS2, OVO, PCNA, PIK3CA, PRDX5, PRPF19, PTGS1, PTGS2, SCN9A, SULT1A1, TNF, TP53, TYR, XDH* and *XRCC1* (Figure 1C).

### 3.2. Histological Structure of Lung and Trachea Tissues in OVA+PM_10_-Induced Mice

**Figure 2:**
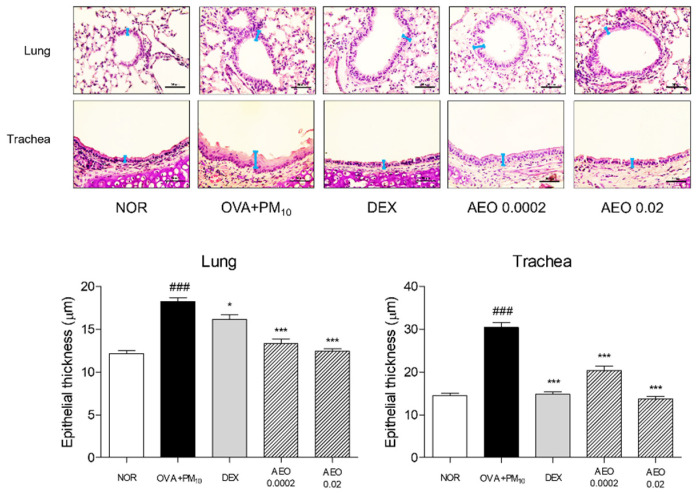
Histological structural changes in lung and trachea tissues stained by hematoxylin and eosin. Magnification is ×400. Blue bars indicate the epithelial thicknesses of lung and trachea tissues, respectively. Results are presented as mean ± standard error of the mean. ### *p* < 0.001 vs. NOR group; * *p* < 0.05 and *** *p* < 0.001 vs. OVA+PM_10_ group.

H&E staining was conducted to demonstrate airway remodeling by lung epithelium thickness. In response to PM_10_ exposure, infiltrated inflammatory cells were detected in the lung epithelium. The lung epithelium was thickened 1.5-fold in the OVA+PM_10_ group compared with non-treated lung epithelium, while the DEX and AEO treatments thinned out the lung epithelium. Especially, treatment with 0.0002% and 0.02% AEO significantly decreased the increases in epithelial thickness in the lung tissues by 26.84% and 31.67%, respectively. In addition, the thickness of trachea epithelium was significantly increased 2.1-fold by OVA and PM_10_ sensitization. AEO inhalation at 0.0002% and 0.02% concentrations by nebulizer dose-dependently prevented the increases in epithelial thickness of trachea tissues by 32.99% and 55.04%, respectively (Figure 2).

### 3.3. Goblet Cell Accumulation in Lung and Trachea Tissues in OVA+PM_10_-Induced Mice

**Figure 3:**
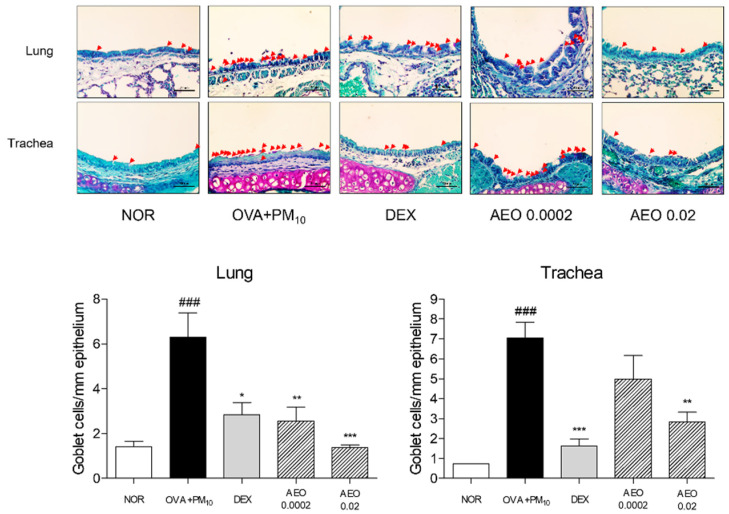
Goblet cell population per area of lung and trachea tissues stained by PAS. Magnification is ×400. Red arrowheads indicate PAS-positive purpled goblet cells. Results are presented as mean ± standard error of the mean. ### *p* < 0.001 vs. NOR group; * *p* < 0.05, ** *p* < 0.01 and *** *p* < 0.001 vs. OVA+PM_10_ group.

PAS staining was conducted to demonstrate goblet cell accumulation. Mucin hypersecretion derived from goblet cell metaplasia was detected by PAS staining, which expressed 4.43-fold PAS-positive goblet cells in the lung tissues of OVA+PM_10_ group compared to the NOR group. Contrary to the OVA+PM_10_ group, the DEX- and AEO-treated groups showed the reduction in PAS-positive cells in the lung tissues. In total, 0.0002% and 0.02% AEO treatments changed the number of PAR-positive goblet cells in the lung tissues by 59.45% and 78.24%, respectively. In addition, the number of goblet cells were markedly increased by OVA and PM_10_ exposure; however, nebulizing DEX and 0.02% AEO in saline reversed that by about 76.96% and 59.70%, respectively (Figure 3).

### 3.4. Collagen Deposition of Lung Tissues in OVA+PM_10_-Induced Mice

**Figure 4:**
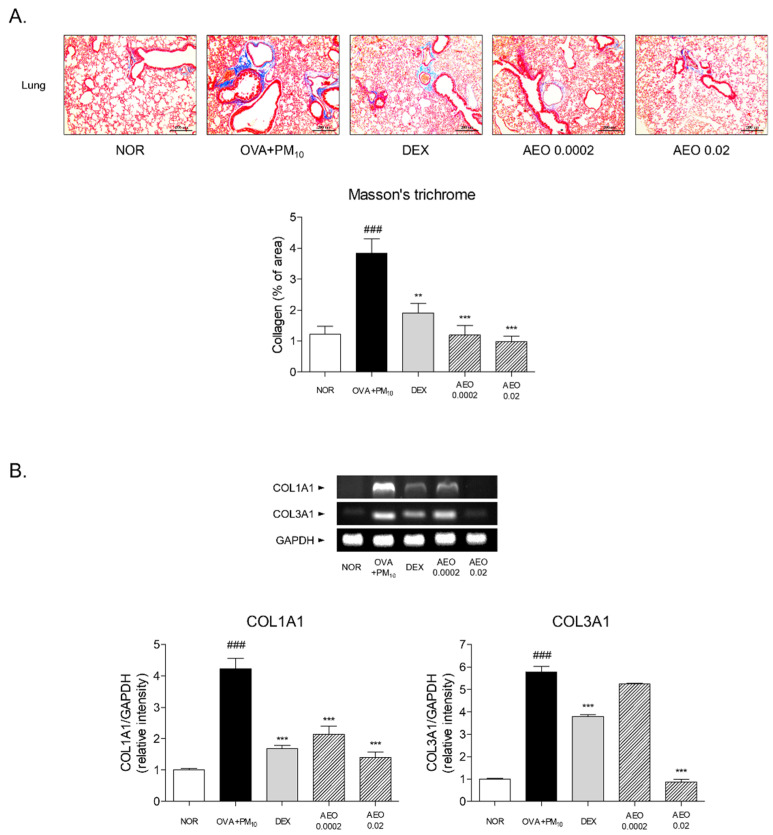
Collagen deposition % of area of lung tissues. (**A**) Lung tissues stained by Masson’s trichrome. Blue-stained area of lung tissues indicates collagen accumulation. Magnification is ×100. Results are presented as mean ± standard error of the mean. ### *p* < 0.001 vs. NOR group; ** *p* < 0.01 and *** *p* < 0.001 vs. OVA+PM_10_ group. (**B**) Expressions of COL1A1, COL3A1 mRNA levels in lung tissues. Results are presented as mean ± standard error of the mean. ### *p* < 0.001 vs. NOR group; *** *p* < 0.001 vs. OVA+PM_10_ group.

The region of interest for collagen deposition and lung fibrosis by exposure to OVA+PM_10_ was quantified through Masson’s trichrome staining. The blue region demonstrated that excessive collagen deposited in the OVA+PM_10_ group by 3.16-fold compared with the non-treated group. DEX treatment showed prevention of collagen deposition stained with blue dye of about 50.40%. In the groups of AEO 0.0002 and AEO 0.02, collagen depositions were apparently reduced by 68.70% and 74.30%, respectively, relative to the OVA+PM_10_ group (Figure 4A).

Considering COL1A1 and COL3A1 as fibrogenic mediators, we determined the effects of AEO on the reduction in collagen deposition at RNA levels. In the lung tissues of the OVA+PM_10_ group, the levels of COL1A1 and COL3A1 were increased 4.21-fold and 5.78-fold, respectively, compared with the lung tissues of the NOR group. DEX treatment reduced the mRNA expression levels of collagen genes, by 60.18% and 34.45%, respectively, in the OVA+PM_10_-exposed mice. Nebulizing 0.02% AEO significantly lowered the levels of COL1A1 and COL3A1 mRNA by 66.90% and 85.06%, respectively (Figure 4B).

### 3.5. Inflammatory Cell Counts in BALF of OVA+PM_10_-Induced Mice

**Figure 5:**
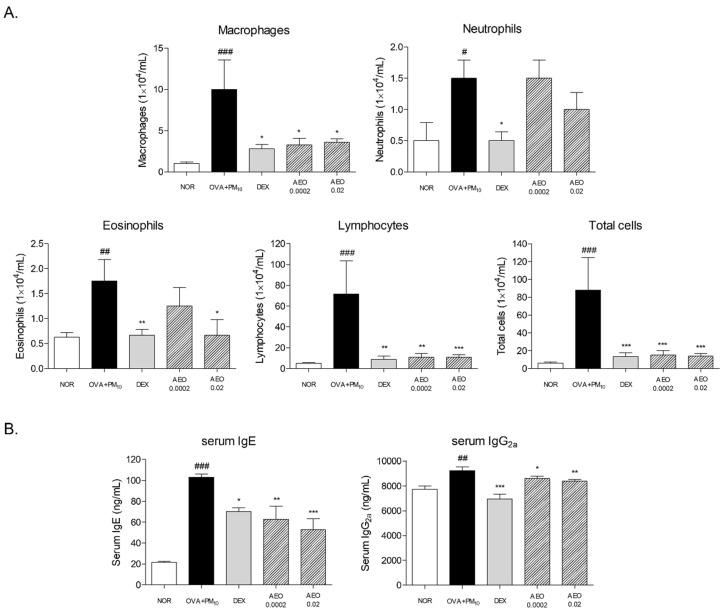
Number of inflammatory cells, including macrophages, neutrophils, eosinophils, lymphocytes and total cells, in BALF (**A**) and serum IgE and IgG2a levels (**B**). Results are presented as mean ± standard error of the mean. # *p* < 0.05, ## *p* < 0.01 and ### *p* < 0.001 vs. NOR group; * *p* < 0.05, ** *p* < 0.01 and *** *p* < 0.001 vs. OVA+PM_10_ group.

The number of cells in BALF from asthmatic mice was remarkably higher than that from normal mice. There were 10-fold, 3-fold, 2.80-fold, 14.90-fold and 14.87-fold increases in macrophages, neutrophils, eosinophils, lymphocytes and total cells in BALF of OVA+PM_10_-induced asthmatic mice. Nebulizing with AEO significantly decreased the number of inflammatory cells in BALF. Increased macrophage numbers were decreased by about 67% and 64% in 0.0002% and 0.02% AEO-treated BALF, while neutrophils were not changed. The number of eosinophils was significantly decreased by 0.02% AEO inhalation up to 61.9%. In addition, there were 85.17% and 85.08% significant changes in the lymphocytes’ number in BALF of 0.0002% and 0.02% AEO-treated mice. Taken together, the increase in total cell numbers was markedly inhibited by AEO treatment at the 0.0002% and 0.02% concentrations of AEO by about 82.61% and 84.20%, respectively (Figure 5A).

### 3.6. Serum IgE and IgG_2a_ in OVA+PM_10_-Induced Mice

There was an excessive increment in serum IgE levels by OVA and PM_10_ exposure by 4.75 times compared to non-treated mice. Treatment of AEO at the 0.0002% and 0.02% concentrations in saline by nebulizer significantly attenuated by about 38.84% and 48.54%, respectively. In addition, a 19.5% increase in IgG_2a_ level was found in the serum of OVA+PM_10_ mice compared to mice of the NOR group. Significant changes in serum IgG2a were observed in the AEO 0.0002% and AEO 0.02% groups (Figure 5B).

### 3.7. Expressions of Pro-Inflammatory and Th2 Cytokines in OVA+PM_10_-Induced Lung Tissues

**Figure 6:**
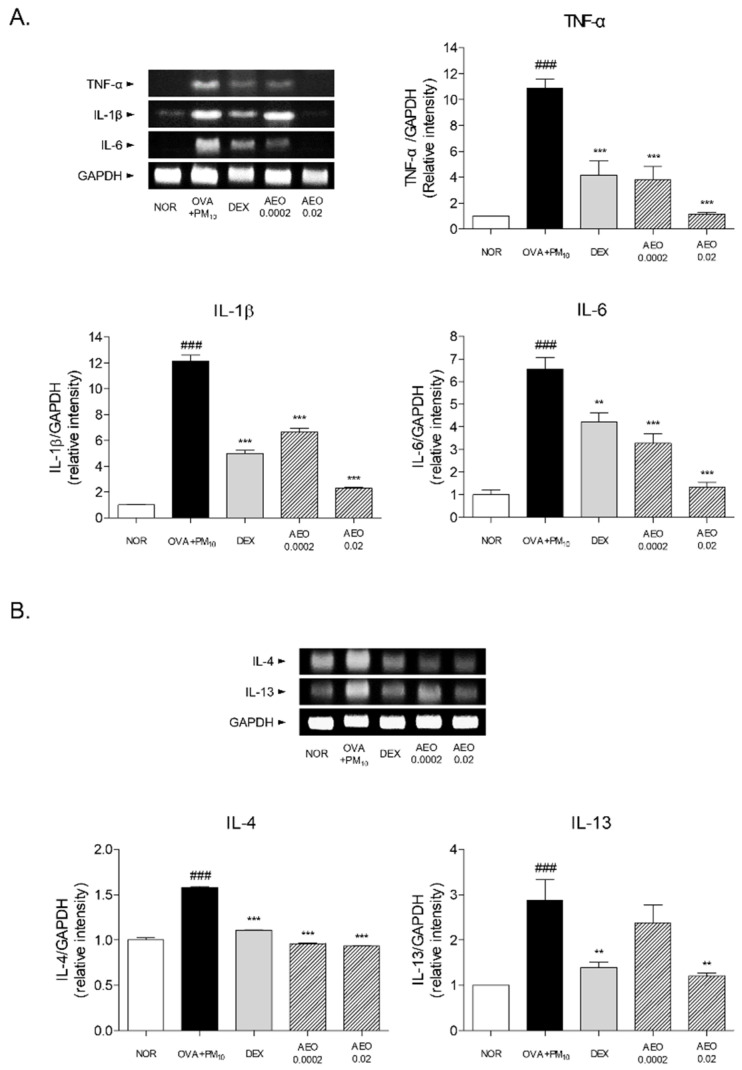
Expressions of pro-inflammatory and Th2-specific cytokines in lung tissues. (**A**) mRNA levels of TNF-α, IL-1β and IL-6 in lung tissues. Results are presented as mean ± standard error of the mean. ### *p* < 0.001 vs. NOR group; ** *p* < 0.01 and *** *p* < 0.001 vs. OVA+PM_10_ group. (**B**) mRNA levels of IL-4 and IL-13 in lung tissues. Results are presented as mean ± standard error of the mean. ### *p* < 0.001 vs. NOR group; ** *p* < 0.01 and *** *p* < 0.001 vs. OVA+PM_10_ group.

To evaluate the anti-inflammatory effects of AEO, the expression levels of pro-inflammatory cytokines and Th2-specific cytokines were investigated in OVA+PM_10_-induced mice. The mRNA expression levels of TNF-α, IL-1β and IL-6 were up-regulated by 10.86-fold, 12.15-fold and 6.56-fold, respectively, in the OVA+PM_10_ group compared to the NOR group, while the DEX group was down-regulated compared to OVA+PM_10_ group, by 61.59%, 58.97% and 35.89% of TNF-α, IL-1β and IL-6, respectively. In the lung tissues of nebulized AEO groups at the 0.0002% and 0.02% concentrations, there were significant changes in the expression levels of TNF-α by 64.86% and 89.55% compared with the OVA+PM_10_ group. The expressions of IL-1β in the AEO 0.0002 and AEO 0.02 groups were markedly decreased by 45.34% and 81.21%, respectively. Moreover, 50.06% and 79.91% decreases in IL-6 mRNA expressions appeared in the AEO 0.0002 and AEO 0.02 groups compared to the OVA+PM_10_ group (Figure 6A).

By measuring the expression levels of Th2 cytokines, we analyzed the reduction in fibrotic effects of AEO in OVA+PM_10_-induced mice. IL-4 and IL-13 levels were significantly increased in the OVA+PM_10_ group compared to the NOR group. The administration of DEX reduced the levels of IL-4 and IL-13 compared to the OVA+PM_10_ group, by 51.53% and 58.05%, respectively. In addition, similar to the DEX group, nebulizing AEO at 0.0002% and 0.02% significantly suppressed the IL-4 expressions in the lung tissues by 38.27% and 39.93%, respectively. The expression of IL-13 in the OVA-and-PM_10_-exposed lung tissues was markedly attenuated by 66.02% (Figure 6B).

### 3.8. Expressions of MMPs in OVA+PM_10_-Induced Lung Tissues

**Figure 7:**
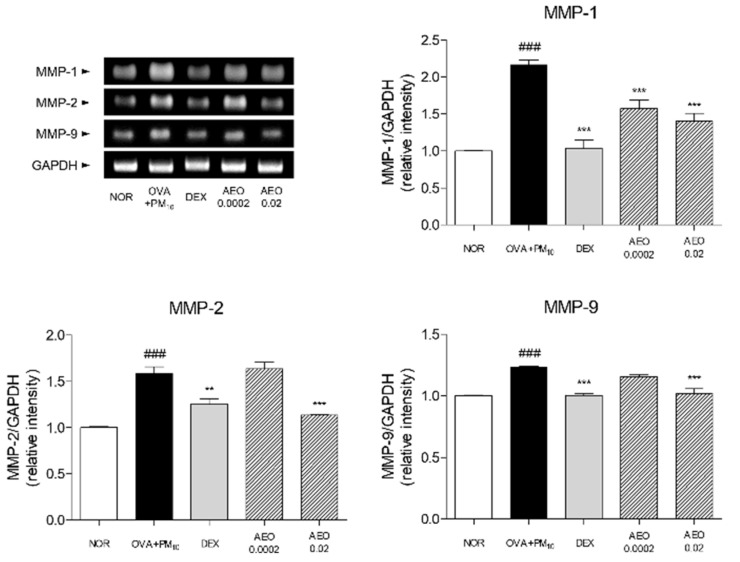
Expressions of MMPs, including MMP-1, -2 and -9, in lung tissues. Results are presented as mean ± standard error of the mean. ### *p* < 0.001 vs. NOR group; ** *p* < 0.01 and *** *p* < 0.001 vs. OVA+PM_10_ group.

The MMP-1, -2 and -9 protein expressions were increased by 2.17 times, 1.59 times and 1.23 times by OVA and PM_10_ sensitization in the lung tissues, respectively. Inhalation of 0.02% AEO by asthmatic mice significantly decreased the expressions of MMP-1, -2 and -9 by 35.32%, 28.48% and 17.27%, respectively (Figure 7).

### 3.9. Expressions of Fibrotic Mediators in OVA+PM_10_-Induced Lung Tissues and PM_10_-Induced A549 Cells

**Figure 8:**
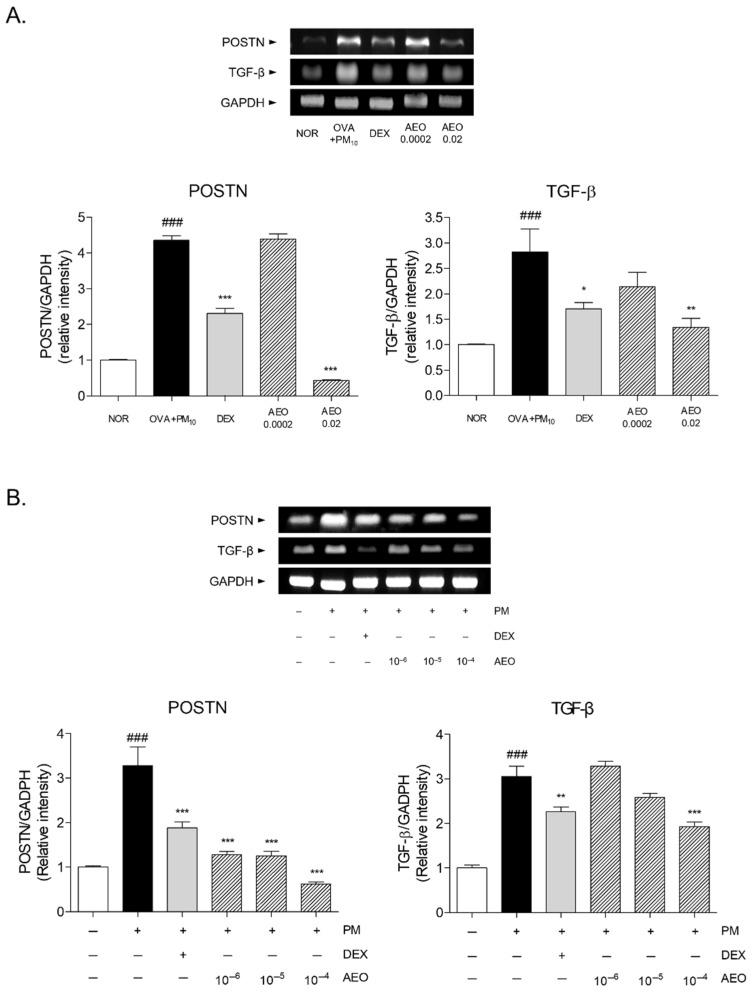
Expressions of fibrotic mediators including POSTN and TGF-β. (**A**) mRNA levels of POSTN and TGF-β in lung tissues. Results are presented as mean ± standard error of the mean. ### *p* < 0.001 vs. NOR group; * *p* < 0.05, ** *p* < 0.01 and *** *p* < 0.001 vs. OVA+PM_10_ group. (**B**) mRNA levels of POSTN and TGF-β in A549 lung epithelial cells. Results are presented as mean ± standard error of the mean. ### *p* < 0.001 vs. non-treated cells; *** *p* < 0.001 vs. PM_10_-stimulated cells.

The POSTN and TGF-β levels were estimated to check whether AEO had an effect on the regression of lung fibrosis in OVA+PM_10_-induced mice. The mRNA expressions of POSTN and TGF-β were significantly increased by 4.35-fold and 2.83-fold, respectively, in the lung tissues of the OVA+PM_10_ group compared to those of the NOR group. In comparison with the OVA+PM_10_ group, the 0.02% AEO treatment by nebulization showed decreased mRNA levels of POSTN and TGF-β in the lung tissues by 90.12% and 52.42%, respectively, compared to the OVA+PM_10_ group (Figure 8A). Furthermore, the levels of POSTN and TGF-β were evaluated in the PM_10_-stimulated A549 lung epithelial cells. PM_10_ induction significantly raised POSTN and TGF-β mRNA levels 3.28-fold and 3.05-fold, respectively, in A549 cells. A549 cells treated with AEO at the concentrations of 10^−6^, 10^−5^ and 10^−4^ (*v*/*v*%) showed 61.06%, 61.76% and 81.13% suppression of POSTN levels. In total, a 36.85% reduction in TGF-β expression was observed in the 10^−4^ AEO-treated cells in the presence of PM_10_ (Figure 8B).

### 3.10. Expressions of Epithelial–Mesenchymal Transition (EMT) Markers in OVA+PM_10_-Induced Lung Tissues and PM_10_-Induced A549 Cells

**Figure 9:**
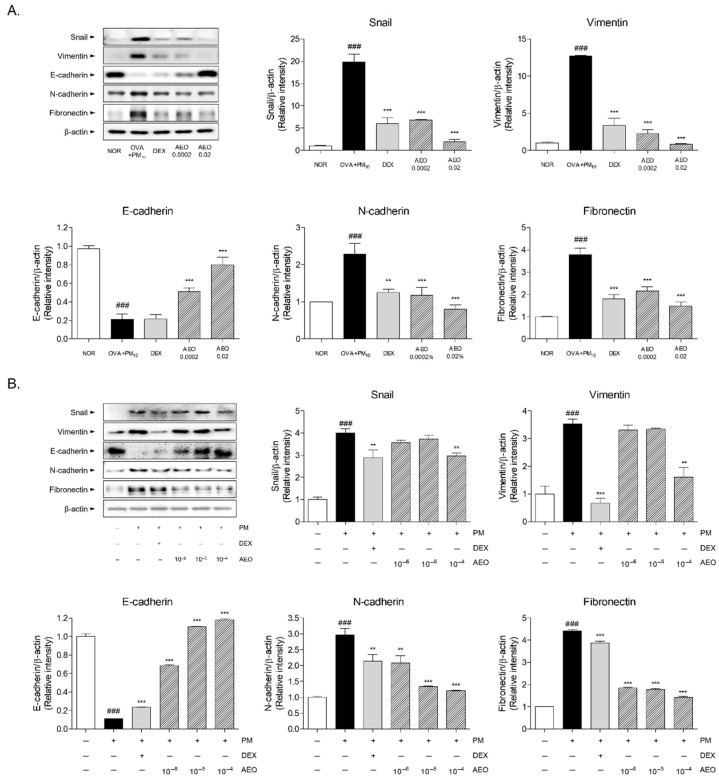
Expressions of epithelial–mesenchymal transition markers including snail, vimentin, E-cadherin, N-cadherin and fibronectin. (**A**) Protein levels of snail, vimentin, E-cadherin, N-cadherin and fibronectin with quantified values in lung tissues. Results are presented as mean ± standard error of the mean. ### *p* < 0.001 vs. NOR group; ** *p* < 0.01 and *** *p* < 0.001 vs. OVA+PM_10_ group. (**B**) Protein levels of snail, vimentin, E-cadherin, N-cadherin and fibronectin with quantified values in A549 lung epithelial cells. Results are presented as mean ± standard error of the mean. ### *p* < 0.001 vs. non-treated cells; *** *p* < 0.001 vs. PM_10_-stimulated cells.

The expressions of transcription factors of EMT, including snail, vimentin, E-cadherin, N-cadherin and fibronectin, were analyzed in both lung tissues (Figure 9A) and A549 lung epithelial cells (Figure 9B). The protein expressions of snail and vimentin were increased 19.8-fold and 12.7-fold by OVA and PM_10_ exposure. There were significant changes in snail expressions by 13.26% and 43.44% in the AEO 0.0002 and AEO 0.02 groups in comparison with the lung tissues of the OVA+PM_10_ group. By pre-treating 0.0002% and 0.02% AEO, the expressions of vimentin were significantly decreased by 82.3% and 93.7% compared to the OVA+PM_10_ group. In PM_10_-stimulated A549 lung epithelial cells, the expressions of snail and vimentin were increased by 3.53 and 4.00 times in comparison with the non-treated cells. In the 10^−4^ *v*/*v*% AEO-treated cells in the presence of PM_10_, there were reductions in snail and vimentin protein levels by 25.90% and 54.46%, respectively. The transition markers E-cadherin and N-cadherin were also down-regulated and up-regulated by 0.11 times and 2.97 times after PM_10_ incubation compared to non-treated cells. Amounts of 10^−6^, 10^−5^, 10^−4^ *v*/*v* % of AEO-treated A549 cells in the presence of PM_10_ showed increases in protein E-cadherin expression by 6.08 times, 9.80 times and 10.49 times, while N-cadherin expressions were significantly decreased by 29.87%, 55.12% and 59.44% in the 10^−6^, 10^−5^, 10^−4^ *v*/*v*% AEO-treated A549 cells with PM_10_. In addition, fibronectin expressions were 3.79 times and 4.40 times increased in OVA+PM_10_-induced asthmatic lung tissues and PM_10_-stimulated A549 lung epithelial cells, respectively. In total, the 0.0002% and 0.02% AEO treatments by nebulizer significantly inhibited the increase in fibronectin expression in the lung tissues by 43.03% and 60.78%. In the lung epithelial cells treated with 10^−6^, 10^−5^ and 10^−4^ *v*/*v*% AEO in the presence of PM_10_, the protein expressions of fibronectin were markedly decreased by 58.27%, 59.72% and 67.96%, respectively.

### 3.11. The Predicted Functional Partners of POSTN by STRING Network

**Figure 10:**
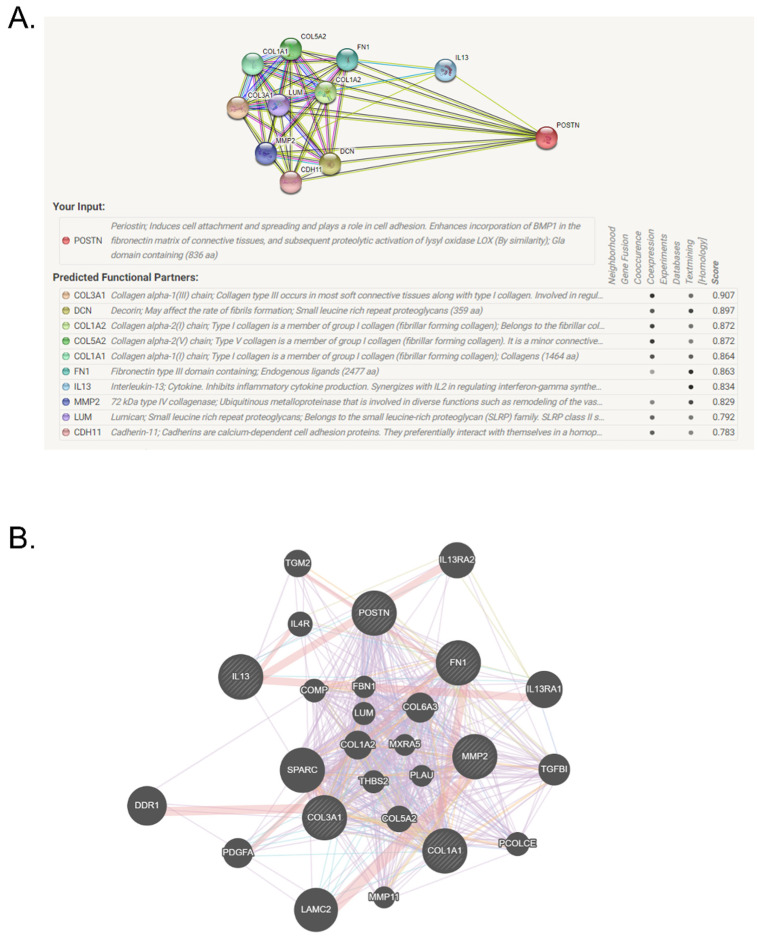
STRING network representing the predicted functional partners of POSTN. Predicted functional partners with POSTN (**A**). Network of POSTN and functional partners (**B**).

To visualize the protein–protein interaction network of POSTN, STRING was employed and the obtained network is shown. The predicted functional partners of POSTN were COL13A, decorin, COL1A2, COL5A2, COL1A1, fibronectin 1, IL-13, MMP-2, lumican and cadherin-11 (Figure 10A). The network of POSTN and functional protein partners was constructed (Figure 10B).

## 4. Discussion

Asthma, a representative disease of respiratory diseases, is a chronic disorder characterized by airway inflammation, hyperresponsiveness and remodeling [23]. Increasing evidence indicates that PM can increase the risk of developing asthma and exacerbate existing asthma. According to previous research, PM_10_ inhalation into the airways can promote inflammation, immune response, oxidative stress and airway remodeling in the respiratory tracts via the production of proinflammatory and inflammatory cytokines [24,25]. Considering the recent use of essential oils from natural products to treat respiratory diseases, we constructed a gene set network of AEO and compared it to the asthma-related genes to demonstrate the association between asthma disease and AEO inhalation. As a result, 48 targeted genes of AEO among 100 whole genes were found to be genes intersecting with asthma ones, with a 48% high matching score. Based on the prediction from the network analysis, an OVA-and-PM_10_-treated in vivo murine model and a PM_10_-treated alveolar epithelial in vitro model were established, to identify the inhibiting effect of the AEO treatment against asthma, demonstrating the therapeutic potential of relieving asthma.

Airway remodeling is closely relevant to various pulmonary diseases, including asthma [26]. In asthma, repeated inflammatory responses have been reported to induce airway structural changes, including airway wall thickening, epithelial cell hyperplasia, goblet cell metaplasia, excessive deposition of the ECM, proliferation of airway smooth cells and accumulation of myofibroblasts, leading to epithelial alterations and subepithelial fibrosis [26]. In addition, air pollution is known to be one of the fatal mediators for airway remodeling and mucus hyperproduction in asthma [27]. Histological changes in the process of airway remodeling induced by OVA and PM_10_ intervention were observed in nebulizing AEO-treated mice. In the lung tissues of the OVA-and-PM_10_-treated models, there were significant pathological histological changes, such as thickening of epithelial cells in the airways and lungs, metaplasia of mucin-secreting goblet cells and excessive collagen deposition by AEO nebulization for 5 min/day for a total of 44 days, indicating that AEO has a property to prevent airway structural changes through the trachea to the lung in asthma. The accumulation of collagen with fibrogenic mediators including COL1A1 and COL3A1 in the lung tissues was significantly inhibited by AEO inhalation. In addition to collagen expression, MMPs, another major ECM component, contribute pathologically to airway remodeling by ECM deposition and degeneration [28]. It was reported that MMP-2 and MMP-9 contribute to tissue remodeling, which occurs by impaired balance between the deposition and degradation of the ECM and cellular migration of the epithelial cells, fibroblast, neutrophils, lymphocytes and dendritic cells [29]. There was a predominant upregulation of COL1A1, COL3A1 and MMP-1, -2 and -9 mRNA expression in the lungs of the experimental groups treated by OVA and PM_10_. Suppression of these upregulated factors by AEO nebulization further supplements the anti-pulmonary fibrogenic effect of AEO.

In the development of asthma, airway remodeling is known to contribute to airway inflammatory responses associated with Th2-specific cells. Especially, PM_10_ sensitization in the airways also provokes an acute immunological and inflammatory response, initiating an influx of inflammatory cells into BALF and airway wall [30]. Representatively, the eosinophil, a major cell type activated by TNF-α, has a key role to mediate pulmonary inflammation and promote airway remodeling by producing IL-13, resulting in mucus hypersecretion in asthma [31]. A recent study suggests that pulmonary TNF-a, IL-1β and IL-6 action can affect airway functioning in asthma [32]. Moreover, it contributes to producing TGF-β, a main fibrotic mediator involving fibroblast differentiation and production of collagen and other ECMs [33]. As reported by some studies about the correlation with the cellular characteristics of BALF and asthma [34], the numbers of total cells, macrophages, neutrophils, eosinophils and lymphocytes of BALF were significantly decreased by the AEO treatment in the OVA-and-PM_10_-treated asthmatic mice. IgE has been known to be involved in the Th2-like allergic immune response [35]. Moreover, repeated allergen exposure forms and activates IgG and affects allergic airway inflammation [36]. In this study, AEO treatment attenuated the elevation of proinflammatory cytokines, TNF-a, IL-1β and IL-6, in the asthmatic lung tissues. In addition, Th2-specific cytokines, IL-4 and IL-13, in the lung tissues were markedly decreased by AEO inhalation. AEO treatment against exposure to OVA and PM_10_ improved the rapid increase in serum IgE and IgG2a levels. Given the fact that AEO inhibits the production of pro-inflammatory and Th2 cytokines, as well as immunoglobulin-containing IgE and IgG, it is predicted that AEO has therapeutic effects on asthma by inhibiting the Th2-associated immune response.

To verify the mode of action of the AEO inhalation on airway remodeling and inflammation in asthma, various molecules related to airway fibrosis were analyzed. Pulmonary fibrotic responses are characterized by remodeling of the airway, especially lung epithelial structure and excessive accumulation of the ECM [37]. The proportions of collagen, which is a major component of the ECM, vary widely between tissues and especially type I and III collagen are mostly associated with mesenchymal interstitium in the lung [38]. POSTN, a member of the matricellular proteins produced by fibroblasts in the extracellular matrix and airway epithelial cells, has a key role in the maintenance and repair of the structural matrix of the airway and lung by increasing proliferation, collagen deposition and development of collagen fibers and promoting EMT in epithelial cells [39]. POSTN was both suggested to be a biomarker of type 2 immune responses, such as asthma, and to be representing tissue remodeling or fibrosis [40]. In addition to POSTN, TGF-β is a major profibrotic mediator that promotes myofibroblast differentiation, EMT and airway remodeling [41]. POSTN can upregulate TGF-β activation, which promotes collagen production by fibroblasts, leading to the initiation of the EMT [42]. In the present study, AEO treatment attenuated TGF-β overexpression, along with POSTN down-regulation in asthmatic lung tissues and PM_10_-stimulated A549 lung epithelial cells.

Based on previous literature reporting on the contribution of the EMT to pulmonary fibrotic diseases [43], POSTN/TGF-β-induced EMT signaling molecules were further investigated. The transition of E-cadherin to N-cadherin in epithelial cells has been regarded to be responsible for their loss of their function and proteins, called the ‘cadherin switch’ [44]. Epithelial cells undergo EMT and then synthesize the ECM components such as fibronectin and collagen type 1, after which it changes to the mesenchymal phenotype. Tight junction and adherents junction are gradually destructed by activating the transcriptional mediators, including snail and vimentin [45]. The protein expressions of the cadherin-switching factors, E-cadherin/N-cadherin, with snail, vimentin and fibronectin were measured for the prevalence of the EMT and for revealing the inhibitory effect of the AEO treatment on EMT. The OVA-and-PM_10_-treated group showed the downregulation of E-cadherin and the upregulation of N-cadherin, snail, vimentin and fibronectin, which means that the process of EMT occurred. The AEO treatment inhibited the down- and up-regulation of EMT markers, showing that AEO treatment had the capacity to prevent the transformation to mesenchymal features and pathological changes in the airway wall. Following the networks generated by the STRING database analysis using the genes and proteins regulated by POSTN, functional partners related to the cell adhesion and EMT signaling pathway were predicted in silico. The interactions were scored based on the Coexpression and Textmining (Homology). Based on those results derived from the network analysis, tight junctions and adherents junctions, which are involved in pulmonary lung fibrosis, including COL, fibronectin and MMP, were down- or up-regulated in the AEO-treated groups. Taken together, AEO decreased the POSTN and TGF-β expressions in the asthma provoked by allergens and then cadherin switching was promoted by POSTN/TGF-β with the co-expression factors, snail and vimentin. Eventually, collagen deposition in the lung was markedly attenuated by AEO. Given the results from the network prediction and experimental evaluation, the down-regulation of POSTN/TGF-β by AEO treatment might be a key therapeutic target of asthma and lung fibrosis.

## 5. Conclusions

In this study, AEO showed anti-inflammatory and anti-fibrotic effects on asthma linked to air pollution. Based on the network pharmacological analysis, it was confirmed that 48% of the AEO-containing target genes matched with the asthma-related genes. AEO treatment suppressed airway remodeling, including airway thickening, goblet cell metaplasia and collagen deposition. Inflammatory cell numbers in BALF, IgE and IgG_2a_ in serum, as well as expressions of cytokines related to the Th2-mediated response, were reduced by AEO treatment. Additionally, AEO treatment inhibited EMT and fibrotic changes, regulating various cytokine and gene expressions, especially POSTN and TGF-β. In summary, AEO improved the histological appearances including airway inflammation and collagen accumulation in the lung with the decreases in inflammatory cytokines and adhesion molecules. Through the predicted functional partner genes and proteins in silico, the attenuation of POSTN/TGF-β by AEO treatment might regulate cadherin switching, following the decreases in fibrotic mediators. We suggest that the AEO nebulizing treatment can be an alternative therapeutic option for asthma. Further research would be required for confirming the usefulness of the nebulizing treatment by comparing it to other administration methods and further molecular modes of action.

## Data Availability

The data used to support the findings of this study are available from the corresponding author upon reasonable request.

## Supplementary Materials

The following supporting information can be downloaded at: https://www.mdpi.com/article/10.3390/pharmaceutics14030558/s1. Figure S1: Identification of AEO using a GC-MS, Figure S2: Morphological changes in cell structure and shape by incubation of PM_10_ and AEO, Figure S3: Cell viability of A549 cells by treating AEO at the concentrations of 10^−6^, 10^−5^ and 10^−4^ *v*/*v*(%).
