# Supplementary material for: Exploring the Potential Effects and Mechanisms of Asarum sieboldii Radix Essential Oil for Treatment of Asthma
Source: Pharmaceutics. 2022 Mar 3;14(3):558. doi: 10.3390/pharmaceutics14030558 (PMC8953372; doi:10.3390/pharmaceutics14030558)
Supplement: Supplementary file 1 [file pharmaceutics-14-00558-s001.zip › pharmaceutics-1570983-supplementary.pdf]

pharmaceutics

Supplementary Materials: Exploring the Potential Effects and Mechanisms of *Asarum sieboldii* Radix Essential Oil for Treatment of Asthma

Jae Min Han, Mi Hye Kim, La Yoon Choi, Gyeongsang Kim, Woong Mo Yang

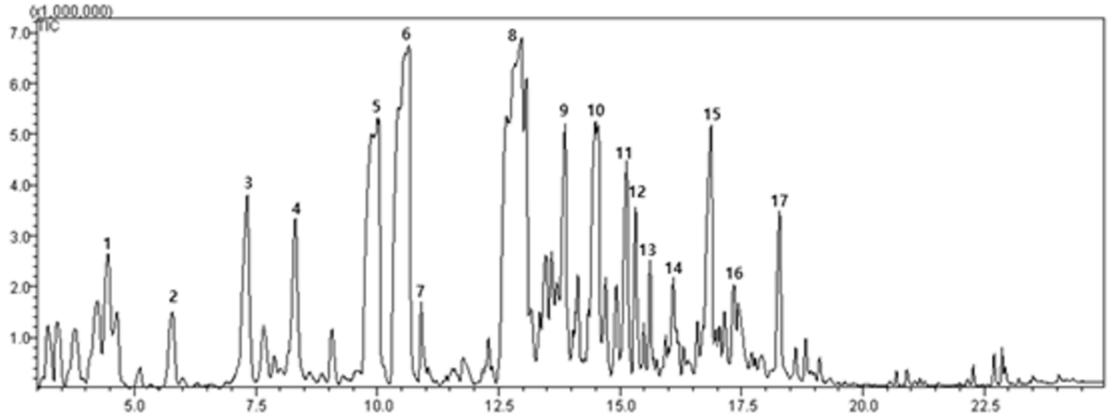

Figure S1. Identification of AEO using a GC-MS.

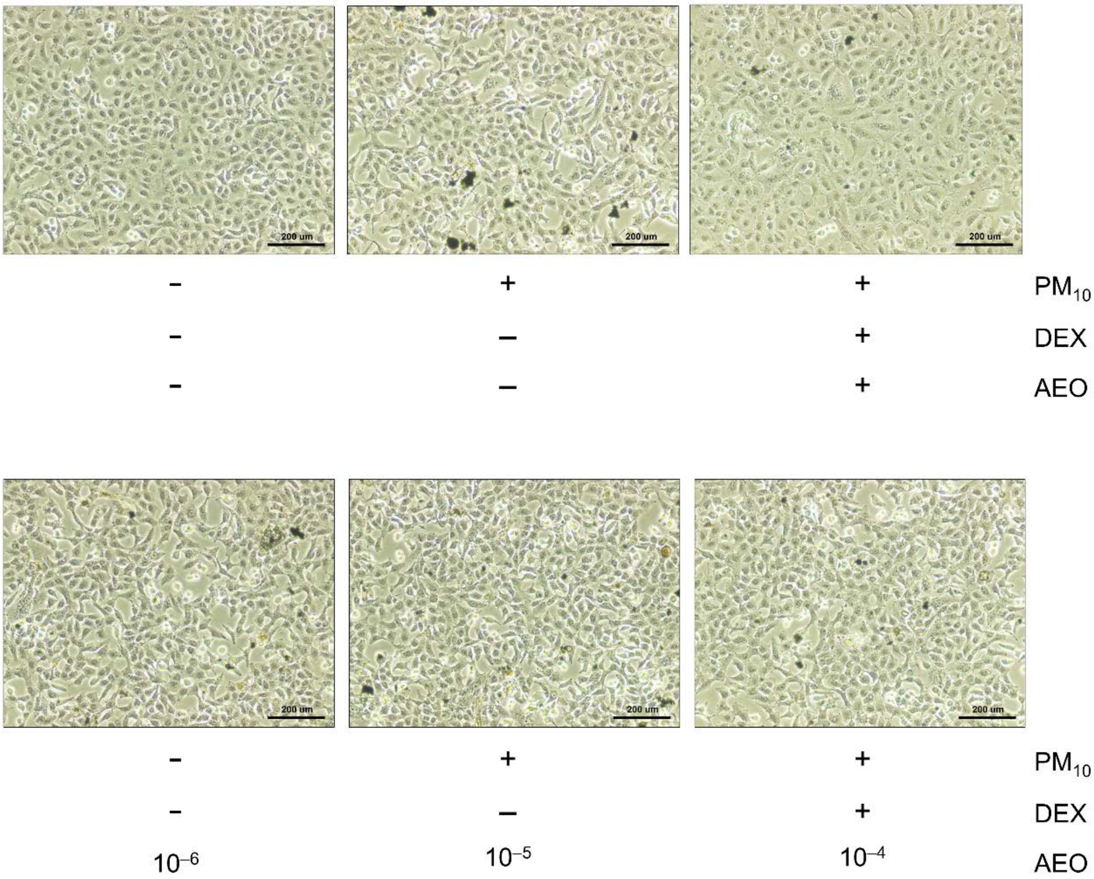

Figure S2. Morphological changes in cell structure and shape by incubation of PM_{10} and AEO.

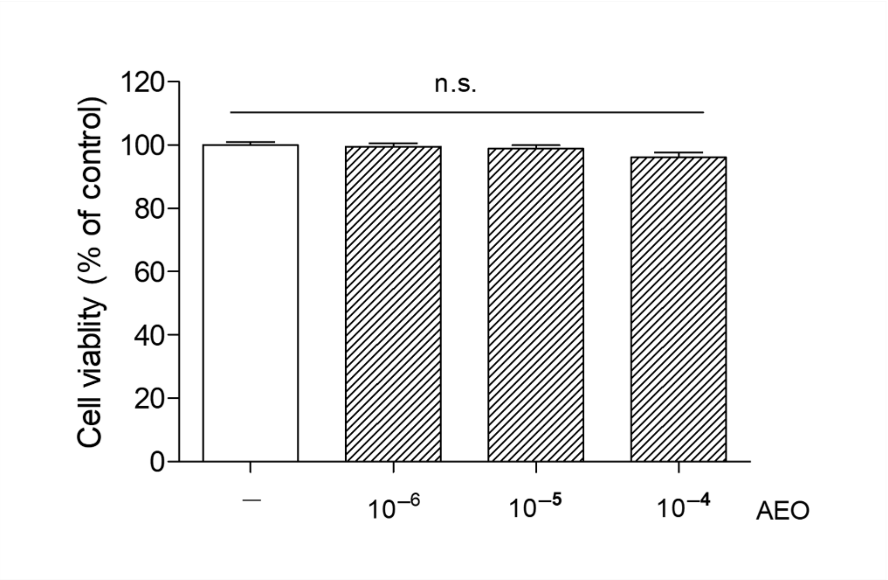

Figure S3. Cell viability of A549 cells by treating AEO at the concentrations of 10^{-6}, 10^{-5} and 10^{-4} *v*/*v*(%).
